# The Impact of COVID-19 Related Lockdown on Dental Practice in Central Italy—Outcomes of A Survey

**DOI:** 10.3390/ijerph17165780

**Published:** 2020-08-10

**Authors:** Bruna Sinjari, Imena Rexhepi, Manlio Santilli, Gianmaria D′Addazio, Piero Chiacchiaretta, Piero Di Carlo, Sergio Caputi

**Affiliations:** 1 Department of Medical, Oral and Biotechnological Sciences, University “G. d′Annunzio” Chieti-Pescara, 66100 Chieti, Italy; imena.rexhepi@gmail.com (I.R.); manlio.santilli@alumni.unich.it (M.S.); gianmariad@gmail.com (G.D.); scaputi@unich.it (S.C.); 2 Electron Microscopy Laboratory, University “G. d′Annunzio” Chieti-Pescara, 66100 Chieti (CH), Italy; 3 Department of Neuroscience, Imaging and Clinical Sciences, University “G. d′Annunzio” Chieti-Pescara, 66100 Chieti, Italy; piero.chiacchiaretta@gmail.com; 4 CAST, Center of Advanced studies and Technologies, University “G. d′Annunzio” of Chieti-Pescara, 66100 Chieti, Italy; piero.dicarlo@unich.it; 5 Department of Psychological, Health & Territorial Sciences, University “G. d′Annunzio” Chieti-Pescara, 66100 Chieti, Italy

**Keywords:** SARS-CoV-2, COVID-19, dentistry, contagion

## Abstract

The COVID-19 pandemic has affected lives and professions worldwide. We aimed to determine the behavior of dentists during the lockdown in Central Italy through an online survey. We demonstrated that the most frequent of urgencies, not otherwise manageable through telemedicine, was dental pulp inflammation. Although a statistically significant increase in the use of some of the personal protective equipment (PPE) from pre to during lockdown was shown, dentists were afraid of being infected during the dental procedures. Moreover, we showed that digital dentistry, telemedicine, use of the rubber dam, distancing of the appointments and further structural changes at the dental office are necessary to reduce the contagion among dentists and patients. No significant differences were shown between gender.

## 1. Introduction

The evolution of the diffusion of the new virus, called “SARS- CoV-2”, counts, to date, high numbers of infected people, and this needs to be monitored due to [1,2] the very high contagiousness and the main transmission routes described. The latter are direct, human-to-human (as caused by coughing, sneezing, droplets of saliva expelled during the phonation) or indirect by contact with a contaminated surface and then touching the main body mucous membranes such as oral, ocular and nasal [3,4]. In a recently published paper, it was shown that, especially during the COVID-19 outbreak, dental practice can represent a high risk of contagion [5]. In fact, the aerosols generated by dental procedures, the smallest droplets suspended in the air, as well as the direct passage between patients and operators can all contribute to spread the infection [5]. The biological risk of transmitting SARS-CoV-2 when performing dental procedures is extremely high due to the presence of aerosol particles of saliva, blood and secretions [6]. In addition, this aerosol/droplets production increases the contamination of dental equipment, instruments and surfaces [7,8].

On the other hand, dental emergencies are definitely possible also for patients affected with COVID-19 and in these cases, contact cannot be avoided [7,9]. The Italian Government declared the lockdown on 9 March 2020 and several European countries followed this example. However, there were no restrictions regarding dental clinical practice, but indications were given to reduce activity to urgencies.

In Italy, dental activity was recognized as a service of primary necessity by the ministerial decree of 22 March 2020 [10] and during the COVID-19 pandemic, dental practice was limited to urgency and emergency treatments which could not be postponed. The National Federation of the Order of Surgeons and Dentists (FNOMCeO) [11], Commission of the Order of Dentists (CAO), the National Association of Italian Dentists(ANDI) [12] and several other scientific dental societies have produced recommendations on dental activity regulating patients′ triage and patients′ management before and after dental treatment.

The management of acute dental emergencies and urgent dental care is essential to reduce the number of hospital admissions due to the consequences of these clinical conditions [13]. Indeed, with the suspension of routine dental care, more patients than usual may need to be hospitalized for the management of acute dental infections that spread to the respiratory tract and require intensive care [13,14]. On the other hand, dental practitioners could follow patients digitally using tools such as telemedicine to ensure patient safety and minimize repeated patient contact [15]. Moreover, the risk of COVID-19 transmission from healthcare providers to patients is quite high and it is therefore necessary to implement proactive and preventive measures to contain the spread of the virus [16]. On 2 August 2020, the Italian region of Abruzzo recorded over 3.389 positives to Sars-Cov-2 cases compared to 248.070 recorded in Italy [17].

Thus, the aim of this survey was to investigate dentists′ attitudes and perceptions regarding the COVID-19 outbreak and to analyze which dental emergencies/urgencies mostly occurred during the Sars-CoV-2 pandemic lockdown in the Abruzzo region, in the center of Italy.

## 2. Materials and Methods

### 2.1. Study Design

An online structured survey composed of 45 questions was sent to dental practitioners affiliated with the National Association of Italian Dentists (ANDI) section of the Abruzzo region, in order to investigate dentist behavior and to analyze their reactions in relation to Sars-CoV-2 pandemic restrictive measures introduced by the Italian national administrative order of 10 March 2020 (DM-10M20) [10]. The survey focuses mainly on a specific geographical area, the Abruzzo region, located in the center of Italy. This region has 4 provinces: Chieti, Pescara, (the two relevant areas of our academic institution), Teramo and L′Aquila.

During the lockdown period in Abruzzo, from 9 March to 4 May 2020, more than 3000 total cases were recorded compared to 211,938 total cases recorded in Italy. The total number of hospitalized patients was 831 and 332 deaths [18] were recorded. The survey was administered through the contact lists of the regional and local Italian Dental Association (ANDI) and a webinar. Thus, it was sent to 885 dentists in the area and to 300 participants during a webinar for dentists on 17th April, 2020. The survey was created using the free-access Google Forms application and the link to the online survey was sent by email. This survey was divided into five sections as shown in detail in Table A1.

### 2.2. Ethical Consideration

Participants provided their informed consent in accordance with the EU General Data Protection Regulation GDPR (UE) n. 2016/679 before beginning the survey. Data collection took place in the time period from 8 April to 1 May 2020. The survey was submitted in an anonymous way through the contacts of the ANDI members list and through the webinar. Thus, it was not necessary to have the ethical approval being it anonymous and, moreover, it did not contain personal sensitive data.

### 2.3. Statistical Analysis

Some of the answers were codified as dichotomous variables, namely as Yes/No responses, or in general as categorical variables, when a multiple-choice selection was requested. Given the nature of our survey, we performed descriptive statistics for most of the questions. For each question, we calculated the percentage of respondents that gave a particular answer with respect to the number of the total responses to the question.

Moreover, a Pearson correlation coefficient (R) was used to investigate the correlation between the age of the participants and some possible influencing factors: the use of telemedicine; the use of the phone triage; diffusion of the digital dentistry; the use of rubber dam; and fear for the future and for being infected. All statistical comparisons were conducted with a significance level of *p* < 0.05. Furthermore, the use of facial masks and other PPEs prior and during the pandemic was compared through the use of the χ2 test, to assess if there was a statistical significance.

The statistical analyses were performed using the GraphPad version 8 (GraphPad Software 2365 Northsides Dr. Suite 560 San Diego, CA 92108) statistical software.

## 3. Results

**Figure 1:**
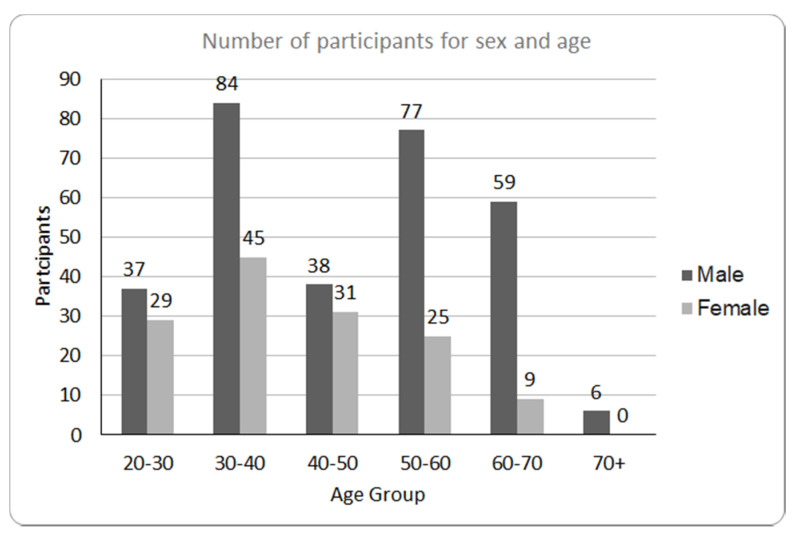
Demographic characteristics of the participants grouped by sex and age.

The total number of dentists who had the possibility to complete the questionnaire was 1185, whilst only 440 completed it (37% of the total) as shown in Figure 1. A total of 68.4% were male, with the majority aged between 30 and 40. A large number of dentists (226; 51.4%) reported to work in the Italian region of Abruzzo, while 15.9% in the southern region of Apulia.

One of the most important pieces of data was that 69.5% (305) reported that they managed dental emergencies recognized by the ADA (American Dental Association) and among these conditions, cellulitis or a diffuse soft tissue bacterial infection with intraoral or extraoral swelling that may potentially compromise the patient′s airway (57.13%) was the most common emergency managed. Meanwhile, among the procedures recognized by the ADA as urgent dental care, dentists reported to have encountered urgent treatments such as severe dental pain from pulpal inflammation (223 = 50.7%); final crown/bridge cementation if the temporary restoration was lost, broken or causing gingival irritation (166 = 37.7%); abscess, or localized bacterial infection resulting in localized pain and swelling (147 33.4%); and tooth fracture resulting in pain or causing soft tissue trauma (128, 29.1%). In fact, after the lockdown was declared in Italy, dentists reported that they performed in 38.4% of cases both dental emergencies and urgent care, in 26.6% of cases only urgent dental care and at least they provided no dental care in 26.4% of cases.

In addition, 68.2% of participants admitted being afraid of being infected by SARS-CoV-2 after performing such emergency/urgent dental procedures during the lockdown period. The Pearson correlation permitted to understand if this variable could be related to the age of the participants. Specifically, it demonstrated that there was no correlation between age and fear of contagion (R = 0.004; *p* = 0.92, ns).

**Figure 2:**
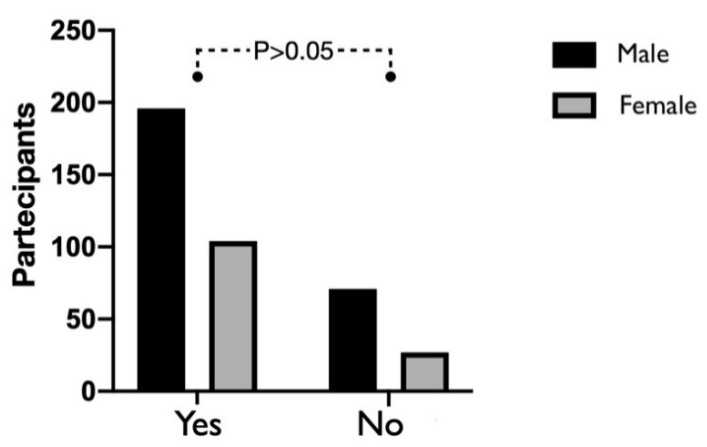
The graph represents the perception of fear of being infected between males and females. The χ2 test showed no statistically significant differences between sex (n.s., *p* > 0.05).

Moreover, by investigating a possible difference according to the gender of the participants (M/F), no statistically significant difference emerged in the sample (*p* = 0.21) (Figure 2).

**Figure 3:**
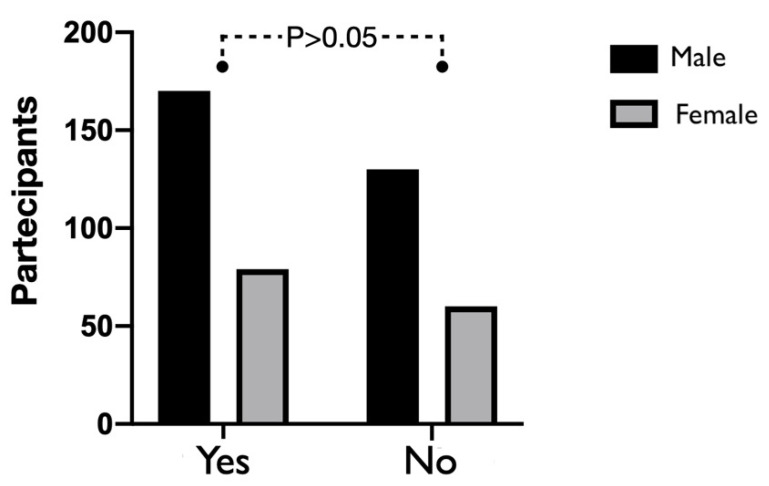
Graphical representation concerning the use of phone triage in the dental office. No statistically significant differences were shown between males and females using the χ2 test (n.s., *p* > 0.05).

Before 10 March 2020, 56.6% of dental offices performed telephone triage to define the real need for emergency treatment and to investigate the risk of exposure to SARS-CoV-2. No statistically significant difference emerged between males and females (*p* > 0.05), as shown in Figure 3. Additionally, 79.5% of them reported that their teams received specific training on how to wear, remove and dispose of any PPE to be used according to the World Health Organization (WHO).

**Figure 4:**
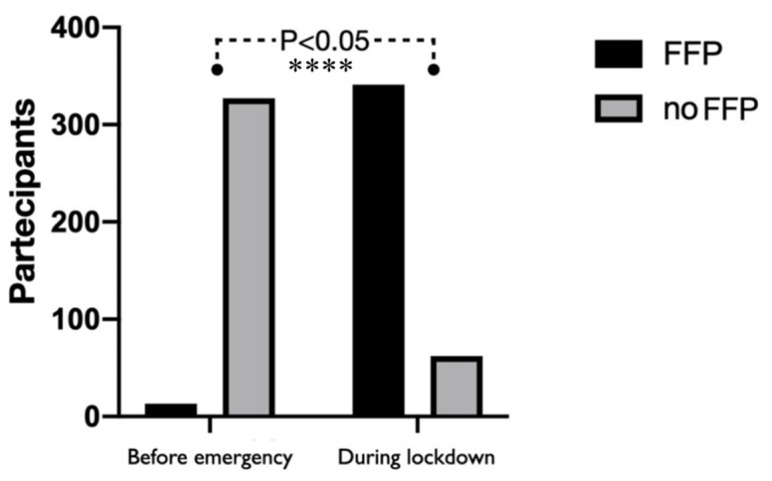
Comparison of filtering face piece (FFP) mask use before and during lockdown. The χ2 test showed highly statistically significant differences (**** *p* < 0.001).

Interesting is the percentage of respondents who, prior to the SARS-CoV-2 breakout, (250, 56.8%) did not use the rubber dam, and even 84.8% of them believed that in the future it could be useful during dental treatments. In this case, an inverse correlation was shown between use of the rubber dam and age, with a statistically significant difference between under 40 and over 40 years old (R = −0.26; *p <* 0.0001). Moreover, routine use of PPE before the Sars-CoV-2 pandemic was asserted by 77.3% of the survey participants and the most PPE used were surgical mask (395, 96.4%), safety glasses (62.2%) or visor (39.4%) and multipurpose cloth headset (41.3%). However, they also admitted that they had to increase the use of PPE during the COVID-19 pandemic (91.6%) and the most PPE used during this period were: safety visor (343, 83.1%), disposable gown (339 = 82.1%), disposable headset (321 = 77.7%), surgical mask (69% = 285) and filtering face piece 2 (FFP2) (62.2% = 257). A statistically significant increase in the use of PPE from pre to during lockdown was shown through the use of the χ^2^ test (*p* < 0.001) as shown in Figure 4.

Additional interesting data were that only 45.75% of dental practitioners believed that digital dentistry could be used more often after the Sars-CoV-2 pandemic breakout. Meanwhile, concerning telemedicine, 36.8% of the interviewees do not consider it valid and only 12.3% have previously used it. Regarding the latter, a correlation was shown between the use of telemedicine and participants’ increasing age. In fact, the over 40 years old participants think that telemedicine should be increased in the future (R = −0.006; *p* = 0.19). On the contrary, a moderate correlation was demonstrated when comparing the different ages and the belief in digital technologies. Definitely, the higher the age, the higher the belief in digital technologies that could help during future emergency moments like the COVID-19 pandemic (R = 0.13; *p* = 0.004).

Finally, the majority of the interviewees (66.6%) reported apprehension about their professional future. However, no statistically significant differences were shown in the correlation of age and future perspectives of dentists (R = 0.008; *p* = 0.86).

## 4. Discussion

The aim of this survey was to understand what type of treatment was provided the most, as well as to thoroughly investigate the perception of the professionals and their behavior during the lockdown period.

Precisely, due to the high risk for dentists discussed by many authors [19,20], the government advised dentists to provide only emergency or urgent services. By analyzing our data relating to the number of operators who performed urgent services, as well as the type of urgency performed, the endodontic treatments resulted as predominant. In fact, 50% of the interviewees went to the dental office during the lockdown for pulp-related problems. This result is actually in line with a recent study which reported that 50.26% of the dental emergencies performed at the department of the School and Hospital of Stomatology at Wuhan University were endodontic [21]. The authors showed that the number of total emergencies was extremely small compared to the same period in 2019 and 2018 [21]. Unfortunately, we did not have the opportunity to compare these data to the previous years at the same period. However, these data suggest that the government restrictions adopted to contain the infection could have forced the population not to go to the dentist even in a dental emergency situation.

In addition, only a small part (3.2%) of dentists interviewed through this survey declared that they went to the dental office predominantly for traumatism. This datum is lower than the global incidence of dental trauma described in the literature. In fact, a systematic review realized by Azami-Aghdash et al. in 2015 reports that among adolescents, traumatism has an average incidence of 17.5% [22]. This higher general percentage—compared to the data presented herein—can be influenced by the reduction in aggregation and sports activities [22]. In fact, traumatic events that occurred at home, at school and during sports activity were more related to dental trauma.

Other surveys have been conducted to analyze the dental population in the pandemic period, but they have different objectives with respect to the present one. Specifically, in April 2020, Ahmed et al. [23] investigated the perception of the state of anxiety and fear in the dental population regarding the risk of contagion. In their study, 78% of the 669 participants were anxious and scared by the devastating effects of COVID-19 [23]. A large number of dentists (90%) were aware of recent changes in the treatment protocols [23]. Similarly, 68.2% of respondents to our questionnaire were affirmed to be afraid of being infected by SARS-CoV-2 after performing urgent dental procedures during the lockdown period. The higher percentage in Ahmed et al.′ s work could be justified by the heterogeneity of the sample selected, referring to 30 different countries that were effected in various ways by the pandemic.

Further, Duruk et al. [24] in April 2020 conducted a survey in order to understand what kind of precautions were taken by the dental population in Turkey. Even in this scenario, more than 90% were frightened by the possibility of contagion, thus confirming the direct role of the dentist and the high risk of contagion [24]. The authors declared that 12% of the participants use N95 masks [24]. On the other hand, in our study, the variation in the type of devices used has been analyzed. The analysis started from 395 dentists (96.4%) who used only the surgical mask before the emergency, during normal dental practice. Only 257 professionals (62.2%) reported to use the FFP2 after the start of the pandemic. These differences can be justified by the difficulty in the intensive wear of N95 masks. In fact, Scarano et al. in a recent study showed that N95 respirators are able to induce an increased facial skin temperature with greater discomfort compared to surgical masks [25].

In Italy, to the authors’ best knowledge, three surveys have been conducted [26,27,28] regarding dentists’ fear of contagion. Anyway, all authors of the abovementioned surveys agree regarding the level of fear and awareness of dentists. However, in May 2020, Consolo et al. [26] focused on psychological aspects and on the diffusion of preventive measures such as the use of N95-type masks and confirmed how the national and international guidelines have been fully implemented by the dental population.

On the other hand, Cagetti et al. [27] in May 2020 mainly investigated the development of pathology, the positive and symptomatic cases, in the dental population. The authors declared that 502 (14.43%) participants had suffered one or more symptoms referable to COVID-19. Moreover, 31 subjects were positive to SARS-CoV-2 virus and 16 subjects developed the disease out of a total of 3599 [27]. Moreover, De Stefani et al. in May 2020 made a questionnaire filled out by 1500 dentists from all parts of the country, analyzing the dentists′ idea and attitudes regarding the risks associated with COVID-19 [28]. In order to reduce the movement of patients, the telephone triage process was highly recommended [29]. Among the dentists interviewed, it emerged that 56% of respondents performed telephone triage to manage emergencies, already before 10 March 2020. Other authors suggest the use of triage in order to minimize the number of patients accessing dental practice by telephone, recognizing the deferrable and non-deferrable services [30]. In addition, among the ministerial recommendations, the use of disinfectants and mouth-rinses with chlorhexidine or not was strongly recommended to reduce the microbial load. [29,31]. Disinfection protocols and professional oral hygiene can also aid in the long-term maintenance of implant supported restorations [32].

Moreover, the use of the appropriate personal protection equipment (PPE), strict dressing and operational protocols were recommended [29]. In this regard, 84.8% of respondents believe that the use of the dental dam can be useful during dental practice to limit the spread of aerosol and aerogenic infections. In fact, since the beginning of the emergency, the use of the dental dam has been strongly recommended [8]. These results are in accordance with other authors who agree on the use of the rubber dam during dental practice to reduce possible transmission of Sars-Cov-2. An interesting result of this study is that 56.8% of dentists did not use the rubber dam before the coronavirus emergency. This datum appears quite worrying, considering the high scientific evidence of the effectiveness of the rubber dam [33,34]. On the other hand, there are many scientific articles in the literature which have declared low values regarding the use of the rubber dam [35]. Another study conducted on dentists from Saudi Arabia confirmed that 21.6% of professionals claim to use the rubber dam [36], whilst in India, about 94% of the subjects were conscious of using it. [37].

Another recent survey conducted on 669 participants from 30 different countries showed that 84% did not use rubber dam isolation for every patient [23]. Considering the possibility of reducing aerosol and the effectiveness of the dam in endodontic and restorative therapies [38], the use of this tool should be extremely encouraged. The results show that the population seems to have received this information, given that 84.8% report to be ready to increase its use. As mentioned, also the use of PPE has undergone major changes as for the rubber dam. In fact, in the pre-COVID period, 96.4% of dentists used only the surgical mask. However, after the emergency, 62.2% declared that they were using the FFP 2. This is what has been suggested by national and international guidelines [29].

On the other hand, a recent review confirmed that, today, there is no convincing evidence that medical masks are inferior to N95 respirators to protect healthcare professionals from laboratory-confirmed viral respiratory infections during routine care and non-aerosol-generating procedures [39].

Along with masks, also the approach through disposable clothing and other disinfection technologies seems to be changed. The disposable gown was used by 31.6% before the emergency and its use reaches 82.1% during the COVID period. Furthermore, 368 operators (88.5%) consider—following the pandemic—the possibility of renewing indoor air either by opening windows or using mechanical ventilation between every patient′s visit, a valid measure to limit the Sars-Cov-2 virus spread. From the analysis carried out, it emerged that most of the participants (88.9%, 391 people) report that the cost of personal protection devices has increased. In light of this, there is still much hesitation about the resumption of clinical activity, both by operators and patients. This feeling of fear and uncertainty can also be highlighted by the results reported through our questionnaire. In fact, when there were still no official guidelines and not much was known about the reopening of dental practices, dentists saw the general situation with concern.

More specifically, on a scale from green to red (where green means optimistic and red pessimistic), 66.6% of doctors surveyed see the future of dentistry from an economic and professional point of view—yellow, whilst 25% of the dentists are pessimistic (red) for the upcoming years and only 8.7% see the future of dentistry in a positive way (green).

## 5. Conclusions

In conclusion, the analyzed data revealed that most professionals respected the advice given, by carrying out only treatments deemed non-deferrable emergencies during the lockdown period in Central Italy. Moreover, being dental pulp inflammation one of the most frequent performed treatments during lockdown, the use of the rubber dam should be mandatory to reduce the risk of contagion in pandemic era. 

Additionally, telemedicine and triage could be useful tools to assess patients′ conditions before the dental visit. In the future, it can helpful in critical situations for the management of dental emergencies not only in a pandemic but also in other cases.

## Appendix A

**Table A1:** All of the survey divided into five sections.

**Section 1: Demographic Data**	**Answers**
1. Informed consent	yes
	no
2. Age	<30
	30–40
	40–50
	50–60
	>60
3. Gender	Male
	Female
4. Dental practice in:	Abruzzo
	Others (specify)
5. Educational qualification	Medicine and Surgery
	Dentistry
	Both
6. Dentistry specialization	yes
	no
7. Which one	Oral Surgery
	Orthodontics
	Pediatric Dentistry
	Maxillofacial Surgery
	Odontostomatology
8. Professional setting	Private Practice
	University Dental Clinic
	Employed in public structure
**Section 2: Dental Procedures Performed During SARS-CoV-2 Lockdown**	**Answers**
9. Do you know the recommendations provided to dentists for the Sars-Cov-2 epidemic by accredited bodies?	Yes
	No
10. From 1 January 2020 to 10 March 2020, you have performed:	All the procedures as usual
	Some type of procedures
	No procedure
11. From 1 February 2020 to 10 March 2020, you have performed:	All the procedures as usual
	Some type of procedures
	No procedure
12. Which of the aforementioned emergency services, as indicated by the American Dental Association (ADA), did you provide performing your professional activity during the lockdown period?	Uncontrolled bleeding
	Cellulitis or a diffuse soft tissue bacterial infection with intra-oral or extra-oral swelling that potentially compromise the patient′s airway
	Trauma involving facial bones, potentially compromising the patient′s airway
	none of the above
	Other
13. Which of the following urgent dental care, as indicated by the American Dental Association (ADA), did you provide performing your professional activity during the lockdown period?	Severe dental pain from pulpal inflammation
	Pericoronitis or third-molar pain
	Surgical post-operative osteitis, dry socket dressing changes
	Abscess, or localized bacterial infection resulting in localized pain and swelling.
	Tooth fracture resulting in pain or causing soft tissue trauma
	Dental trauma with avulsion/luxation
	Dental treatment required prior to critical medical procedures
	Final crown/bridge cementation if the temporary restoration is lost, broken or causing gingival irritation.
	Biopsy of abnormal tissue
	Extensive dental caries or defective restorations causing pain
	Manage with interim restorative techniques when possible
	Suture removal
	Denture adjustment on radiation/oncology patients
	Denture adjustments or repairs when function impeded
	Replacing temporary filling on endo access openings in patients experiencing pain
	Snipping or adjustment of an orthodontic wire or appliances piercing or ulcerating the oral mucosa
	None of the above
	Other
14. The Lockdown in Italy was declared on 9.03.2020. From the following day, you provide:	Dental emergencies and urgent care
	Only dental emergencies
	Only urgent dental care
	No dental care
15. Which of the urgent dental care did you provide most frequently during the lockdown period?	Severe dental pain from pulpal inflammation
	Pericoronitis or third-molar pain
	Surgical post-operative osteitis, dry socket dressing changes
	Abscess, or localized bacterial infection resulting in localized pain and swelling
	Tooth fracture resulting in pain or causing soft tissue trauma
	Dental trauma with avulsion/luxation
	Dental treatment required prior to critical medical procedures
	Final crown/bridge cementation if the temporary restoration is lost, broken or causing gingival irritation.
	Biopsy of abnormal tissue
	Extensive dental caries or defective restorations causing pain
	Manage with interim restorative techniques when possible
	Suture removal
	Denture adjustment on radiation/oncology patients
	Denture adjustments or repairs when function impeded
	Replacing temporary filling on endo access openings in patients experiencing pain
	Snipping or adjustment of an orthodontic wire or appliances piercing or ulcerating the oral mucosa
	None of the above
	Other
16. Which of the dental emergencies did you provide most frequently during the lockdown period?	Uncontrolled bleeding
	Cellulitis or a diffuse soft tissue bacterial infection with intra-oral or extra-oral swelling that potentially compromise the patient′s airway
	Trauma involving facial bones, potentially compromising the patient′s airway
	None of the above
	Other
17. Are you afraid of getting SARS-CoV-2 infection during emergency/urgent dental care?	Yes
	No
	I don′t know
18. Before March 10, 2020, have you ever performed telephone triage in order to investigate for major symptoms of SARS-CoV-2 such as fever, cough, breathing difficulties, muscle pain and sore throat, in patients who asked to be visited?	Yes
	No
19. Has the staff that helps you in clinical practice had specific training on how to wear, remove and dispose of any Personal Protective Equipment (PPE) to be used according to WHO?	Yes
	No
20. In your clinical practice prior to the SARS-CoV-2 epidemic, did you use oral rinses for the patient before any treatment?	Yes
	No
	Sometimes
	Before oral/implant surgery
	Only before professional oral hygiene sessions
21. If you answered yes to the previous question, do you indicate which ones you used most:	Chlorhexidine mouthwashes
	Mouthwashes with essential oils
	Hydrogen peroxide diluted with water
	Others
22. In your clinical practice prior to the SARS-CoV-2 epidemic, did you use the rubber dam for all performance where is its intended use?	Yes
	No
	Not always
23. If you did not always answer the previous question and used, therefore, the rubber dam only for some performances, can you indicate which ones?	
24. If you have not used the rubber dam, do you think that in the future it can be useful to use it in all the treatments that provide for it?	Yes
	No
	I don′t know
**Section 3: The Organization of Dental Services in the Presence of an Epidemic From Sars-Cov-2**	**Answers**
25. To date, are the visit and/or care provided performed with specific PPE?	Yes
	No
26. If your answered yes to the previous question was “yes, can you indicate which of the following PPE were used?	Surgical mask
	FFP2 mask
	FFP3 mask
	Safety glasses
	Safety visor
	Disposable headset
	Multipurpose cloth cap
	Disposable gown
	Overshoes
	Double gloves
	Others
27. Did you use these PPE in your clinical practice prior to the SARS-CoV-2 epidemic?	Yes
	No
28. If your answered yes to the previous question was “yes, please indicate which ones:	Surgical mask
	FFP2 mask
	FFP3 mask
	Safety glasses
	Safety visor
	Disposable headset
	Multipurpose cloth cap
	Disposable gown
	Overshoes
	Double gloves
	Others
29. To date, are patient visits and care provided with further contagion containment measures?	Yes
	No
30. If your answered yes to the previous question was “yes, please indicate which ones:	Disinfection of the support surfaces.
	Casings for instrumentation,
	Particular tools for decontamination.
	Providing patients with hand sanitizer upon arrival.
	Waiting rooms free from potential contaminants.
	Disposable bags to be provided to patients to put their personal belongings.
	Renew indoor air either by opening the windows or using mechanical ventilation, possibly in between patients.
	The delay of the appointments to not saturate the waiting room.
	Other ventilation systems.
	Others.
**Section 4. Economic-Financial Considerations Of The Sars-Cov-2 Epidemic On Dentistry Activities**	**Answers**
31. Is your clinical activity supported by structured employees?	Yes
	No
32. Do you plan to use social safety nets (for example redundancy fund)?	Yes
	No
33. During the period of SARS-CoV-2 epidemic, did you notice an increase in the cost of PPE?	Yes
	No
	I don′t know
34. If your answered yes to the previous question was “yes, please indicate in what percentage:	100%
	90%
	80%
	70%
	60%
	50%
	40%
	30%
	20%
	10%
	I don′t know
35. Since the onset of the SARS-CoV-2 epidemic, your activity has decreased in percentage of:	100%
	90%
	80%
	70%
	60%
	50%
	40%
	30%
	20%
	10%
	I don′t know
36. In your opinion, are the economic and financial support provided for government and social security for dentists sufficient?	Yes
	No
	I don′t know
**Section 5: Feelings/Concerns Regarding Their Professional Future**	**Answers**
37. Do you think that the organizational aspects of your professional life will change after this epidemic?	Yes
	No
	I don′t know
38. Do you think that in the future structural adjustments will be necessary in the places where the profession is carried out to reduce any risk of contagion?	Yes
	No
	I don′t know
39. Do you think that any structural adjustments in the places where the profession is carried out to reduce any risk of contagion will be temporary until the discovery of a cure/vaccine?	Yes
	No
	I don′t know
40. Do you feel adequately protected by sector representative and membership bodies? (ex: associations, order etc.)	No
	Yes, from all representative bodies
	Yes, but only by representative association (ANDI, AIO)
	Only from the national federation of orders of surgical doctors and dentists
	I don′t know
41. Do you believe in telemedicine?	Yes
	No
	Not too much
	I don′t know
42. Have you used telemedicine previously?	No
	Yes
	Few times
	I don′t know
	I think I will use it in the future
43. Do you think that digital dentistry will be more used in the future also following the effects of SARS-CoV-2 epidemic?	Yes
	No
	I don′t know
44. What do you think are the right measures to reduce the negative impact of SARS-CoV-2 in the dental sector?	Lowering the rates of services
	Expanding funding methods to support patients
	Continue to work as before
	Do more information- institutional communication on medicine and oral health
	Increase essential levels of assistance from institutions
	Increase the types of benefits reimbursed by insurance companies
45. How do you see your future work according to a scale where green is for very good and red is for bad/disastrous?	Yellow
	Red
	Green
